# Selective inhibition of histone deacetylase 2 induces p53-dependent survivin downregulation through MDM2 proteasomal degradation

**DOI:** 10.18632/oncotarget.3100

**Published:** 2014-12-31

**Authors:** Sung-Keum Seo, Chang-Sun Hwang, Tae-Boo Choe, Seok-Il Hong, Jae Youn Yi, Sang-Gu Hwang, Hyun-Gyu Lee, Sang Taek Oh, Yun-Han Lee, In-Chul Park

**Affiliations:** ^1^ Division of Radiation Cancer Research, Korea Institute of Radiological & Medical Sciences, Gongneung-dong, Nowon-gu, Seoul, Republic of Korea; ^2^ Human Resource Biobank, Cheil General Hospital, Catholic Kwandong University College of Medicine, Jung-gu, Seoul, Republic of Korea; ^3^ Department of Microbiological Engineering, Kon-Kuk University, Gwangjin-gu, Seoul, Republic of Korea; ^4^ Department of Laboratory Medicine, Korea Cancer Center Hospital, Korea Institute of Radiological & Medical Sciences, Gongneung-dong, Nowon-gu, Seoul, Republic of Korea; ^5^ Division of Radiation Effects, Korea Institute of Radiological & Medical Sciences, Gongneung-dong, Nowon-gu, Seoul, Republic of Korea; ^6^ Department of Microbiology and Immunology, College of Medicine, Yonsei University, Seongsan-no, Seodaemun-gu, Seoul, Republic of Korea; ^7^ Department of Radiation Oncology, College of Medicine, Yonsei University, Seongsan-no, Seodaemun-gu, Seoul, Republic of Korea

**Keywords:** HDAC2, Lung cancer, Mdm2, p53, Survivin

## Abstract

In the present study, we found that selective inhibition of histone deacetylase 2 (HDAC2) with small inhibitory RNA (siRNA) induced survivin downregulation in a p53-dependent manner. Interestingly, suberoylanilide hydroxamic acid (SAHA) or knockdown of HDAC2 induced downregulation of Mdm2, a negative regulator of p53, at the protein level. SAHA and/or HDAC2 siRNA increased Mdm2 ubiquitination, and MG132, an inhibitor of proteosome function, prevented HDAC2 inhibition-induced degradation of Mdm2. Clinically, the mRNA levels of HDAC2 and survivin were prominently overexpressed in lung cancer patients compared to normal lung tissues. Silencing of HDAC2 enhanced the cell death caused by ionizing radiation in lung cancer cells. Collectively, our results indicate that selective inhibition of HDAC2 causes survivin downregulation through activation of p53, which is mediated by downregulation of Mdm2. They further suggest that HDAC2 may exert a dominant effect on lung cancer cell survival by sustaining Mdm2-survivin levels.

## INTRODUCTION

Members of the histone deacetylase (HDAC) family, encoded by 18 distinct genes, are divided into four classes—class I, class IIa, class IIb, class III and class IV—based on their homology. HDACs catalyze the removal of acetyl groups from lysine residues located on amino terminal tails of histone protein [1]. By controlling the level of acetylation of core histones, HDACs are generally associated with repression of transcription and reduced gene expression [2]. In addition to interacting with chromatin proteins, HDACs can lead to altered expression of a large number of genes through direct interaction with non-histone proteins, such as the transcription factors E2F and Stat3, and the tumor-suppressor p53 [3, 4]. Several studies have shown that class I and II HDACs (HDAC1-10) are overexpressed in some cancers, including gastric cancer, colorectal cancer, prostate cancer, and lung cancer [5, 6]. Moreover, both altered expression and mutation of HDACs have been linked to cancer formation and progression, reflecting the fact that these changes in HDACs induce aberrant transcription of key genes that regulate important cellular functions [2]. In light of this, class I and II HDACs have emerged as attractive targets for anticancer therapy. In fact, two recently developed HDAC inhibitors—vorinostat (suberoylanilide hydroxamic acid (SAHA), Zolinza) and depsipeptide (romidepsin, Istodax)—have been approved by the US Food and Drug Administration (FDA) as anticancer drugs [1, 7]. HDAC inhibitors have been shown to induce apoptotic cell death and growth arrest in various cancer cells, promote reactive oxygen species generation, and inhibit angiogenesis through downregulation of genes involved in regulating angiogenesis, including hypoxia-inducible factor 1 alpha (HIF1α) and vascular endothelial growth factor (VEGF) [8]. Suberoylanilide hydroxamic acid (SAHA) has been shown to enhance radiosensitivity in preclinical tumor models [9]. SAHA treatment in combination with ionizing radiation has been reported to attenuate the upregulation of DNA damage-repair proteins, including DNA-activated protein kinase (DNA-PK) and the recombinase Rad51 [10]. Although HDAC inhibitors have been evaluated in clinical trials, the different and specific roles of individual HDACs in carcinogenesis remain unclear.

Survivin, a member of the inhibitor of apoptosis family, is undetectable in most normal adult cells but is frequently overexpressed in a variety of cancer cells. It has been shown that survivin inhibits apoptosis, promotes tumor-associated angiogenesis, and serves as a determinant of resistance to various anticancer therapies [11]. Survivin expression inhibits cell death induced by various apoptotic stimuli *in vitro* and *in vivo* [12]. Notably, overexpression of survivin is detected in early-stage non-small-cell lung cancer patients, suggesting that survivin may play a role in lung tumorigenesis [13]. It has also been reported that survivin gene expression is transcriptionally repressed by wild-type p53, which binds directly to the survivin promoter [14, 15]. As a downstream factor that is highly expressed in cancer and regulated by p53, survivin is a dual mediator of resistance to apoptosis and cell-cycle progression [16]. Thus, regulation of the p53-survivin signaling pathway is important for cell survival. We previously showed that SAHA is a potential therapeutic agent by virtue of its downregulation of survivin in lung cancer [17]. HDAC inhibitors have been shown to induce cell death by suppressing survivin expression in various cancer cells, including non-small cell lung cancer (NSCLC), renal cell carcinoma and epidermoid carcinoma [18–22]. A better understanding of the molecular mechanism underlying the regulation of survivin expression by specific members of the HDAC subfamily and the role of p53 in this process could provide a novel strategy for minimizing toxicity and acquiring high efficacy through targeting of survivin.

In the present study, we investigated the role of individual HDACs in regulating survivin expression. We further explored possible molecular mechanism(s) by which inhibition of HDAC2 negatively regulates survivin expression and elucidated the relationship between inhibition of HDAC2 and radiosensitivity in non-small-cell lung cancer cells. We found that inhibition of HDACs with a chemical inhibitor or genetic knockdown of HDAC2 downregulated survivin by increasing p53 protein stability. Interestingly, the increase in p53 protein induced by HDAC2 knockdown was mediated by proteosomal degradation of the p53 negative regulator, Mdm2. Together, these findings suggest that HDAC2 might be an important molecular player in the regulation of Mdm2 and survivin expression levels in lung cancer cells.

## RESULTS

### SAHA induces survivin downregulation through p53 activation

**Figure 1:**
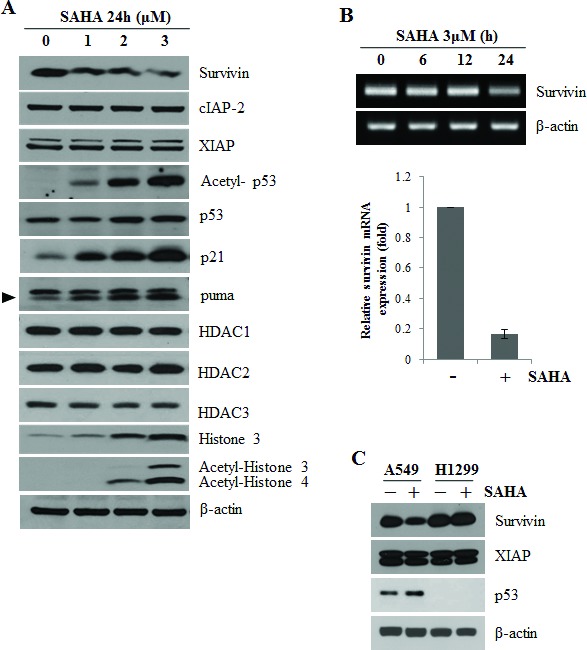

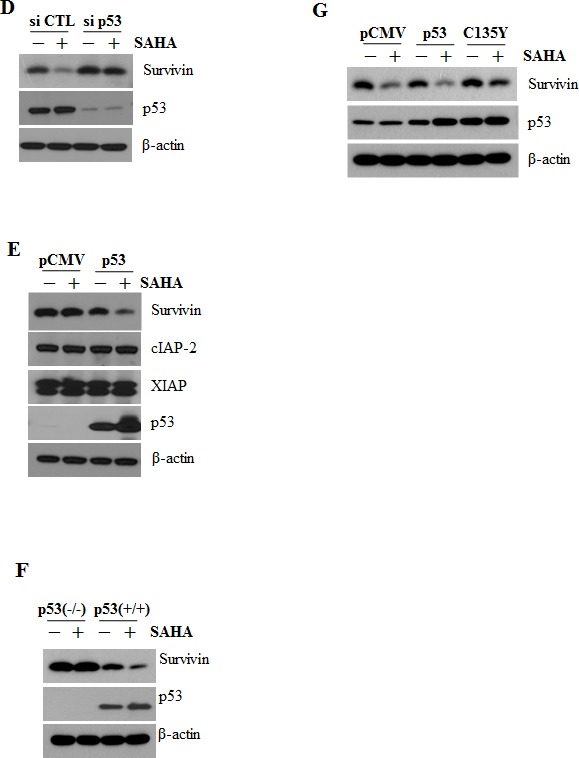
SAHA-induced survivin downregulation by p53 activation After incubation, cells were lysed and analyzed by Western blotting and RT-PCR as described in Materials and Methods. β-actin was used as a control for equal protein and cDNA loading. In qPCR, Survivin mRNA expression levels were determined by the relative to the control groups using 2^−ΔΔCt^ method. Values were represented as means ± SD of three independent experiments. Immunoblots and PCR bands are representative of at least three independent experiments. A. A549 cells were treated with 0–3 μM SAHA for 24 h. B. A549 cells were treated with 3 μM SAHA for various times (RT-PCR) or for 24 h (qPCR). C. A549 and H1299 cells were treated with 2 μM SAHA for 24 h. D. A549 cells were transfected with 50 nM p53 siRNA (si p53) or negative control siRNA (si CTL) and were treated with 2 μM SAHA (+) for 24 h. E. H1299 cells were transfected with 0.1 μg p53 wild-type expression plasmid (p53) or empty vector (pCMV) using Lipofectamine and treated with 2 μM SAHA for 24 h. The specificity of p53 interference or overexpression was confirmed using an anti-p53 antibody. F. HCT 116 colon cancer cell lines, p53(−/−) and p53(+/+) were treated with 2 μM SAHA (+) for 24 h. G. A549 cells were transfected with 0.1 μg p53 wild-type expression plasmid (p53), p53 dominant negative expression plasmid (C135Y, 135C to Y mutation) or empty vector (pCMV) and treated with 2 μM SAHA for 24 h.

In our previous report, we examined the effect of SAHA on the expression of survivin in human non-small-cell lung cancer cells [17]. We found that SAHA decreased the expression of survivin. Here, we confirmed that SAHA induced a concentration-dependent decrease in survivin levels in A549 cells; it also increased acetyl-p53, p21, puma and acetyl-histone levels without expression changes of HDACs (Fig. 1A). RT-PCR analyses showed that survivin mRNA levels were also downregulated by treatment with SAHA for 24 h (Fig. 1B). These results suggest that SAHA regulates survivin expression at the transcriptional level.

To further investigate whether p53 is associated with SAHA-induced downregulation of survivin, we examined survivin expression in p53 wild-type A549 cells and p53-null H1299 cells after treatment with SAHA. SAHA decreased survivin protein levels in A549 cells, but did not affect survivin levels in H1299 cells (Fig. 1C). Furthermore, knockdown of p53 with siRNA significantly attenuated the reduction in survivin protein levels induced by SAHA in A549 cells (Fig. 1D). In H1299 cells transfected with a p53 expression plasmid, SAHA treatment resulted in downregulation of survivin (Fig. 1E). We examined the level of survivin using Western blotting in HCT116 colon cancer cell lines, p53(−/−) and p53(+/+) after treatment with SAHA. In (Fig. 1F), basal survivin level in p53(+/+) cell line are lower than p53(−/−) cell line. p53 expression was increased and survivin expression was decreased by SAHA in p53(+/+) cell line, but SAHA did not affect survivin levels in p53(−/−) cells. Transfection of A549 p53-wild cells with a plasmid expressing the p53 C135Y mutant (C135Y) led to recovery survivin down-regulation induced by SAHA (Fig. 1G). The p53 C135Y expression plasmid encoding a dominant-negative mutant can no longer interact with p53 binding sites because of a conformational change induced by mutation of cysteine 135 to tyrosine [24]. Collectively, these results indicate the p53 activation plays an important role in SAHA-induced survivin downregulation.

### Selective inhibition of HDAC2 induces survivin downregulation

**Figure 2:**
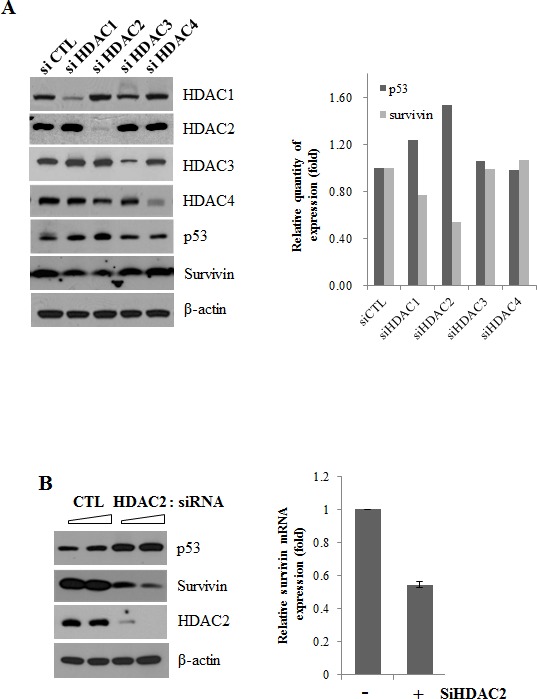

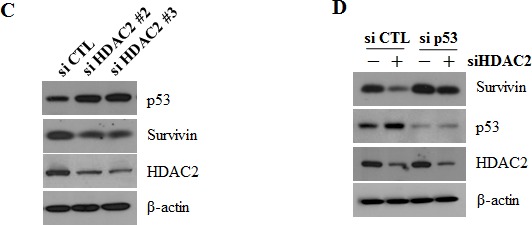
Suppression of survivin expression by HDAC2 siRNA After incubation, cells were lysed and analyzed by Western blotting and qPCR. β-actin was used as a control for equal protein loading. In qPCR, Survivin mRNA expression levels were determined by the relative to the control groups using 2^−ΔΔCt^ method. Values were represented as means ± SD of three independent experiments. Immunoblots are representative of at least three independent experiments. A. A549 cells were transfected with 50 nM siRNA targeting specific HDAC isoforms (si HDAC1, si HDAC2, si HDAC3, si HDAC4) or negative control siRNA (si CTL) and incubated for 24 h. The relative protein level of p53 and survivin are presented by the graph of the quantitative values. B. A549 cells were transfected with 60 or 120 nM HDAC2 siRNA or control siRNA and incubated for 24 h. (Western blotting) and cells were transfected with 60 nM HDAC2 siRNA (+) or control siRNA (−) and incubated for 24 h. (qPCR) C. A549 cell were transfected with two different HDAC2 siRNA (60 nM) for 24h. D. A549 cells were transfected with 50 nM p53 siRNA and 60 nM HDAC2 siRNA, alone or in combination, and incubated for 24 h.

To identify the role of individual HDACs in survivin expression, we transiently transfected A549 cells with siRNA individually targeting the HDAC family members, HDAC1, HDAC2, HDAC3, and HDAC4. Western blot analyses showed that each selective siRNA specifically decreased the protein level of its targeted HDAC. Interestingly, we found that knockdown of HDAC2 changed survivin and p53 protein levels prominently (Fig. 2A).

Next, we tested the role of p53 in HDAC2 siRNA-mediated downregulation of survivin in p53 wild-type A549 lung cancer cells. HDAC2 siRNA induced an increase in p53 protein levels and corresponding reduction in survivin protein levels dose-dependently as well as survivin mRNA levels (Fig. 2B). When we used two different HDAC2 siRNAs, the effect on survivin was in same manner with Fig. 2B. (Fig. 2C) Furthermore, knockdown of p53 with siRNA significantly reversed the HDAC2 siRNA-induced reduction in survivin protein (Fig. 2D). These results indicate that HDAC2, among HDAC isoforms, specifically plays a role on regulation of survivin and p53 acts as a mediator of HDAC2 knockdown-induced survivin downregulation.

### HDAC2 inhibition induces Mdm2 downregulation through proteasomal degradation

**Figure 3:**
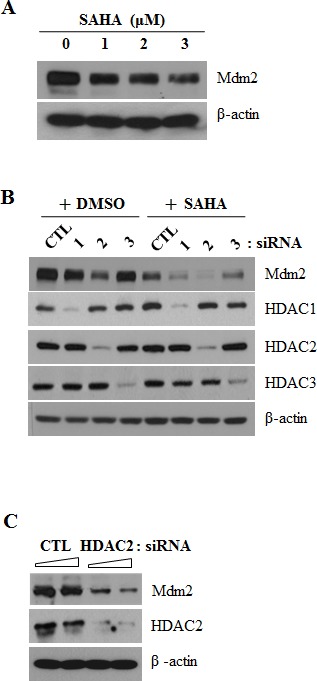

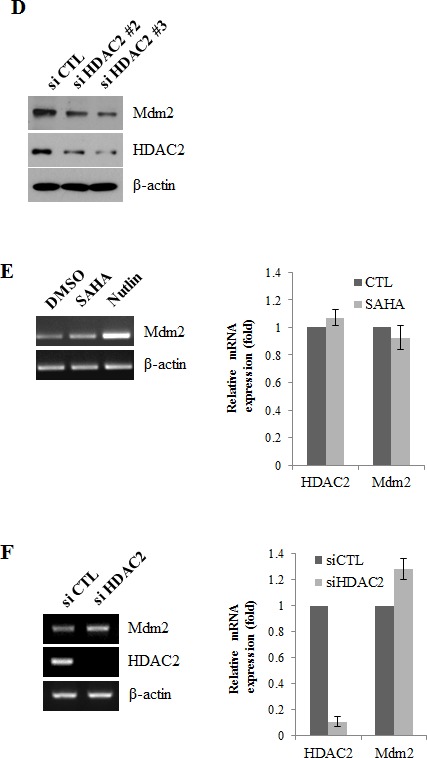
Mdm2 downregulation by SAHA or HDAC2 siRNA After incubation, cells were lysed and analyzed by Western blotting and RT-PCR. β-actin was used as a control for equal protein and cDNA loading. In qPCR, mRNA expression levels were determined by the relative to the control groups using 2^−ΔΔCt^ method. Values were represented as means ± SD of three independent experiments. Immunoblots and PCR bands are representative of at least three independent experiments. A. A549 cells were treated as described for Figure 1A. B. A549 cells were transfected with 60 nM siRNA targeting specific HDACs (HDAC1, HDAC2, HDAC3) or negative control siRNA, and then treated with 2 μM SAHA for 24 h. C. A549 cells were treated as described for Figure 2B. D. A549 cells were treated as described for Figure 2C. E. Cells were treated with 2 μM SAHA or 5 μM Nutlin-3A (positive control) for 24 h and then performed RT-PCR (Left) and qPCR (Right). F. Cells were treated with 60 nM HDAC2 siRNA or negative control siRNA for 24 h and then performed RT-PCR (Left) and qPCR (Right).

To identify the molecular mechanism(s) underlying the activation of p53 induced by SAHA or knockdown of HDAC2, we investigated Mdm2 levels after treatment with SAHA or siRNA targeting HDAC2 in A549 lung cancer cells. Unexpectedly, we found that SAHA induced a concentration-dependent decrease in Mdm2 protein levels (Fig. 3A). In Fig. 3B, 3C and 3D, HDAC2 siRNA similarly induced a marked, dose-dependent decrease in Mdm2 levels; in contrast, siRNA targeting HDAC1 or -3 had no such an effect. To investigate the possible mechanism responsible for SAHA-induced Mdm2 downregulation, we first performed RT-PCR to test the expression of Mdm2 mRNA in SAHA or HDAC2 siRNA-treated cells. Nutlin-3A, used as positive control for Mdm2 mRNA regulation [25], markedly increased Mdm2 mRNA levels, whereas SAHA or HDAC2 siRNA had no effect on Mdm2 mRNA levels (Fig. 3E and 3F).

**Figure 4:**
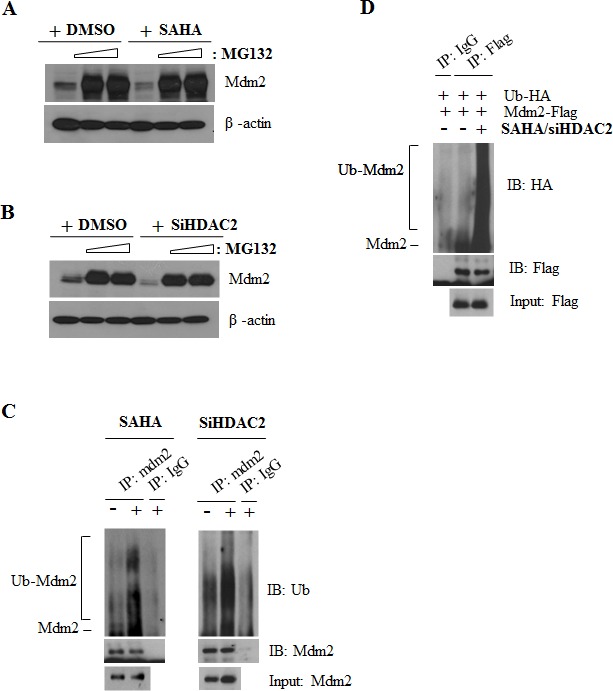
Mdm2 ubiquitination by SAHA or HDAC2 siRNA After incubation, cells were lysed and analyzed by IP and Western blotting as described in Materials and Methods. β-actin was used as a control for equal protein loading. Immunoblots are representative of at least three independent experiments. A. A549 cells were treated with 2 μM SAHA and incubated for 6 h. After incubation, 2.5-5 μM MG132 was added and cells were incubated for an additional 18 h. B. A549 cells were transfected with 60 nM HDAC2 siRNA or control siRNA and incubated for 6 h. After incubation, 2.5-5 μM MG132 was added and cells were incubated for an additional 18 h. C. A549 cells were treated as described for Figure 4A or 4B and then immunoprecipitated using Mdm2 antibody and blotted with anti-Ubiquitin antibody. D. A549 cells were co-transfected with HA-Ub, Flag-Mdm2 plasmid and HDAC2 siRNA and then treated with 2 μM SAHA for 6h. After incubation, 5 μM MG132 was added and cells were incubated for an additional 18h. Cell lysate was subjected to IP assay using anti-Flag antibody and blotted with anti-HA antibody.

These results suggest that Mdm2 is downregulated at the protein level by SAHA. To verify this, we examined SAHA or HDAC2 siRNA effects on Mdm2 protein expression in cells treated with the proteasome inhibitor, MG132. As shown in Fig. 4A and 4B, Mdm2 expression levels were restored in cells co-treated with SAHA or HDAC2 siRNA and MG132. Furthermore, ubiquitination assays confirmed that Mdm2 was ubiquitinated after treatment with SAHA and/or HDAC2 siRNA (Fig. 4C and 4D). These results strongly suggest that inhibition of HDAC2 induces p53-dependent survivin downregulation through proteasome-mediated degradation of Mdm2.

### Correlation between HDAC2 and survivin expression in lung cancer cell lines and overexpression of HDAC2 and survivin in lung cancer patients

**Figure 5:**
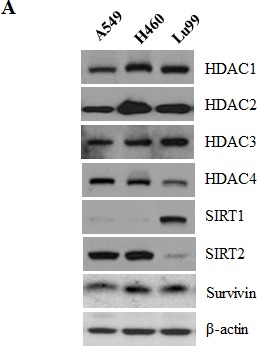

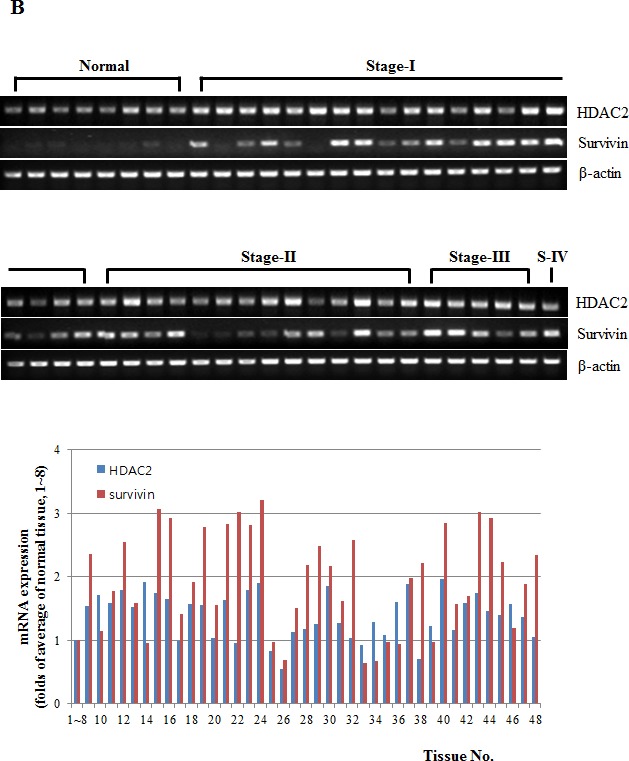
Correlation between HDAC2 and survivin expression in lung cancer cell lines and overexpression of HDAC2 and survivin in lung cancer patients A. A549, H460 and Lu99 cells were lysed and lysates were analyzed by Western blotting. B. The expression of HDAC2 and survivin in lung cancer patient specimens (stage I-20 samples, stage II-14 samples, stage III-5 samples, and stage IV-1 sample) and normal lung tissue (8 samples, total 48 samples). Graphs show the quantitative data of upper bands using Image J program. The relative levels of HDAC2 and survivin mRNA expression in lung cancer patients compared to average of 8 normal lung tissues (set as 1) were represented.

To determine whether HDAC2 and survivin expression are correlated in lung cancer cell lines, we analyzed the expression of HDAC2 and survivin at the protein level in A549, H460 and Lu99 cell lines (non-small lung cancer cell, p53 wild type). As shown in Fig. 5A, survivin expression levels in lung cancer cell lines were highly correlated with HDAC2 expression levels. SIRT1 and SIRT2 are classified to HDAC Class III, and are not inhibited by SAHA. One of the non-histone target of SIRT1, p53, is suggested to play a central mediator of SIRT1-mediated functions in the process of tumorigenesis and senescence. Furthermore, there are new evidences that SIRT1 acts as a tumor suppressor based on its role in negatively regulating beta-catenin and survivin. [26] Therefore, we detected SIRT1 and 2 levels as well as HDAC1-4 in lung cancer cell lines and then confirmed that HDAC2 expression are related to survivin regardless of SIRT1 and SIRT2. Comparison of HDAC2 and survivin mRNA expression levels between normal and cancer were performed using TissueScan Cancer Array (each containing cDNAs from 8 different normal lung and 40 lung cancer patient tissues). In lung cancer patients, survivin and HDAC2 mRNA expression were overexpressed compared to normal lung tissues (Fig. 5B). These results indicate that expression of survivin may be regulated by HDAC2 in lung cancer cells.

### Knockdown of HDAC2 enhances sensitivity to IR-induced cell death

**Figure 6:**
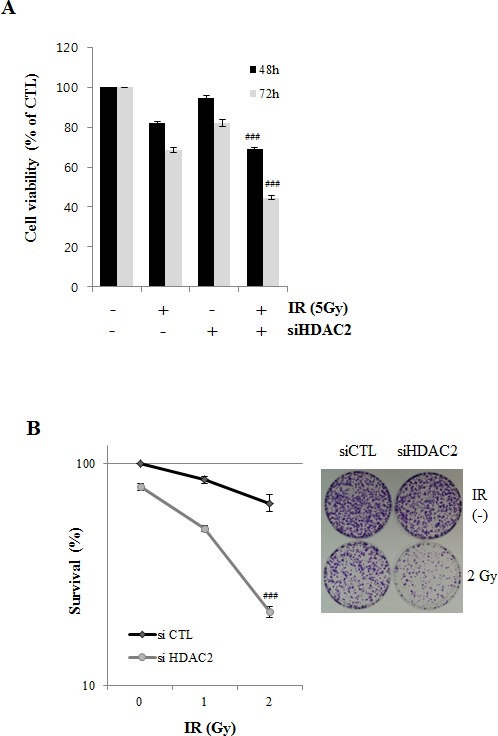

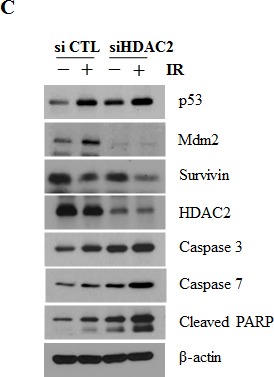

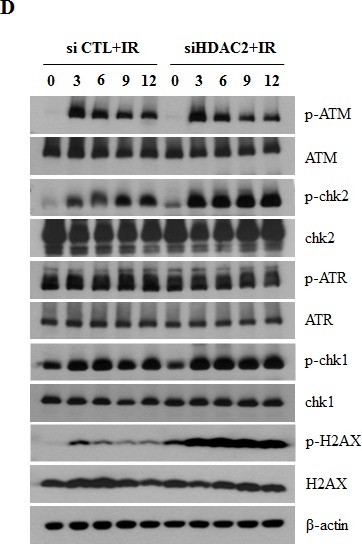

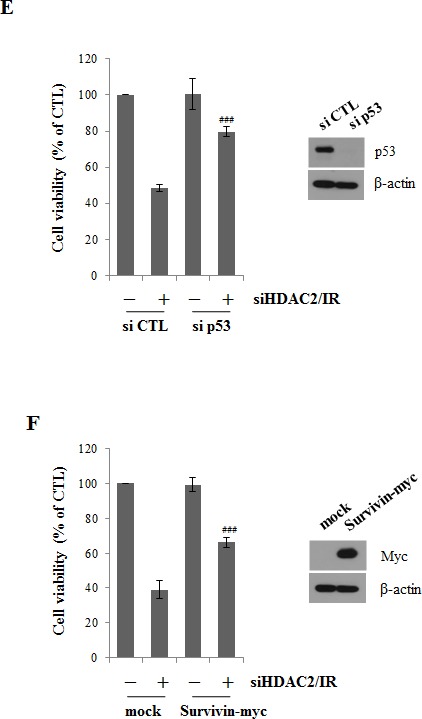

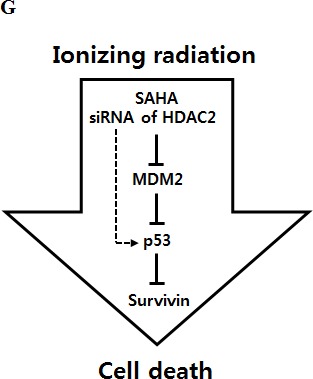
Effect of HDAC2 inhibition on IR-induced cell death After incubation, cells were analyzed by MTT, Western blotting and colony forming assay as described in Materials and Methods. β-actin was used as a control for equal protein loading. Values were represented as means ± SD of three independent experiments. Immunoblots are representative of at least three independent experiments. A. A549 cells were transfected with 60 nM HDAC2 siRNA and then treated with IR (5 Gy) for 48 h or 72h. Cell viability was determined by MTT assay, as described in Materials and Methods, and expressed relative to that of controls (defined as 100%). B. A549 cells were treated with 60 nM HDAC2 siRNA, alone or combination with IR (1 or 2 Gy). After 18 d, colonies were stained and counted. The relative surviving fractions were calculated by dividing the number of colonies in treated cells by that in controls. Each value represents the mean ± S.D. of three independent experiments (^###^*P* < 0.001 vs. IR 2Gy-treated groups). C. A549 cells were treated as described for Figure 6A (48h). D. A549 cells were transfected with 60 nM HDAC2 siRNA. After 6h, then cells were treated with IR. Cells were harvested in time course. E. A549 cells were transfected with 50 nM p53 siRNA and 60 nM HDAC2 siRNA, alone or in combination, and then treated with IR (5Gy) for 72 h. Each value represents the mean ± S.D. of three independent experiments (^###^*P* < 0.001 vs. si CTL/siHDAC2/IR-treated groups). F. A549 cells were co-transfected 0.2 μg survivin-myc plasmid (Survivin-myc) or empty vector (mock) and 60 nM HDAC2 siRNA and then treated with 5Gy IR for 72 h. Each value represents the mean ± S.D. of three independent experiments (^###^*P* < 0.001 vs mock/siHDAC2/IR-treated groups). G. A scheme shows that SAHA or HDAC2 siRNA decreased survivin level through p53-Mdm2 pathway in A549 cells. Downregulated survivin by SAHA or HDAC2 siRNA confers enhanced responsiveness of the cells to ionizing radiation.

Since IR can induce cell death in p53-dependent manner [24, 27], we next determined whether HDAC2 siRNA enhanced the sensitivity of lung cancer cells to IR-induced cell death. As shown in (Fig. 6A), HDAC2 siRNA markedly enhanced the sensitivity of cells to IR-induced cytotoxicity. In colony-forming assays, HDAC2 siRNA had a greater effect on IR sensitivity than negative control siRNA (Fig. 6B). (Fig. 6C) showed that cleavage of caspase 3/7 and PARP were more increased in cells treated with IR and HDAC2 siRNA. These results suggest that antitumor effect of HDAC2 targeting in lung cancer cells might be a apoptotic cell death induced by DNA damage. To find the combined effects of IR and HDAC2 siRNA treatment on ATM/ATR signaling, we examined the levels of phospho-ATM/ATR, -Chk1/2, and -γH2AX after treated with IR or IR/HDAC2 siRNA by Western blotting. As shown in (Fig. 6D), the phosphorylation levels of Chk2 and γH2AX were more increased in cells treated with IR and HDAC2 siRNA than those in IR alone treatment. These results indicate that inhibition of HDAC2 by siRNA enhanced the cytotoxicity which is consequences of the DNA damage induced by IR in lung cancer cells. IR/HDAC2 siRNA-induced cell death was restored in cells transfected with p53 siRNA or survivin overexpression plasmid. (Fig. 6D, 6E) These results suggest that targeting HDAC2 could be effective in the radiotherapy of non-small-cell lung cancers harboring wild-type p53.

## DISCUSSION

The potential role of HDAC inhibitors in downregulating survivin expression has been described previously [18–22]. SAHA, a reversible pan-inhibitor of HDACs, inhibits class I (1, 2, 3 and 8) and II (4, 5, 6, 7, and 9) HDACs. Therefore, to identify which subfamily of HDACs is (are) involved in regulation of survivin, we tested several siRNAs against HDAC1, HDAC2, HDAC3 and HDAC4. The results (Fig. 2 and Fig. 3) show selective depletion of HDAC2 dominantly mediated survivin and MDM2 downregulation. Individual HDACs may play distinct roles and contribute differently in cells. However, they show massive over-compensation and share the link in pathway. In particular, HDAC1 and HDAC2 show compensatory and overlapping functions so that it is complicated to indicate differing effects between specific HDAC subsets [28]. In (Fig. 3B), treatment of HDAC1 knockdown alone inhibited MDM2 to some extent. We thought that it seems to be a compensatory action between HDAC Class I. In this regards, various HDACs subfamily directly or indirectly seems to affect on survivin and Mdm2 expression. In spite of such a compensation between HDACs, siRNA of HDAC2 dominantly downregulates survivin and Mdm2 expression compared with HDAC1 or HDAC3 siRNA. Moreover, p53 expression in protein levels were most remarkably upregulated in cells treated with HDAC2 siRNA other than those of HDACs siRNA. These results suggest that suppression of HDAC2 specifically induced downregulation of survivin through p53 activation in lung cancer cells.

Upon HDAC inhibition, p53 is stabilized and acetylated at lysines 320, 373, and 382 [29, 30]. The intracellular amount of p53 is primarily regulated by the Mdm2 oncoprotein through a negative feedback mechanism, whereby elevated levels of p53 stimulate the expression of Mdm2, which in turn sequesters and ubiquitinates p53, marking it for proteasomal degradation and/or nuclear exclusion [31]. Thus, Mdm2, acting primarily as an E3 ubiquitin ligase, is a key regulator of the p53 tumor suppressor, promoting its degradation and also inhibiting its transcriptional activity by recruiting histone deacetylase and corepressors to p53 [32]. In this context, we examined the role of Mdm2 in the p53-mediated downregulation of survivin induced by inhibition of HDAC2. Interestingly, Mdm2 was downregulated at the protein level by the HDAC inhibitor SAHA and by siRNA targeting HDAC2 (Fig. 3). Consistently with this, ubiquitination assays confirmed that Mdm2 was ubiquitinated after treatment with SAHA and/or HDAC2 siRNA. These results indicate that downregulation of Mdm2 by inhibition of HDAC2 occurred through proteasome-mediated degradation of Mdm2 protein.

It is known that Mdm2 is capable of self-ubiquitination through its E3 ligase function [33]. To test whether self-ubiquitination was responsible for the proteosomal degradation of Mdm2 induced by HDAC2 inhibition, we co-transfected H1299 cells with HDAC2 siRNA and expression constructs for p53 and an E3 ligase-deficient Mdm2 mutant. We found that Mdm2 was decreased by HDAC2 siRNA, suggesting that Mdm2 self-ubiquitination is not involved in the Mdm2 downregulation induced by HDAC2 inhibition (Data not shown). Thus, fully elucidating the regulation of p53 by HDAC will require additional studies to identify the E3 ligase(s) responsible for Mdm2 degradation in this pathway.

In this study, we found that expression levels of survivin were significantly correlated with HDAC2 expression levels in p53 wild type lung cancer cell lines although cases are not sufficient (Fig. 5A). And survivin and HDAC2 expression levels are mostly overexpressed in cancer patients compared to normal lung tissue (Fig. 5B). In this study, we suggest that not only survivin downregulation plays an important role in HDAC2 inhibition-induced cell death, but targeting of the HDAC2 and survivin is the cancer selective treatment. Survivin is rarely present in normal tissue or cells. Increased expression of survivin and HDAC2 are detected in cancer cells including lung cancer [13]. In addition, normal cells are relatively resistant to HDAC inhibitor-induced cell death [8]. HDAC inhibitor can alter the structure and function of a broad range of proteins regulating cell proliferation, migration, and death that are substrates of HDACs. Cancer cells generally have multiple defects in proteins regulating cell proliferation, cell migration, and cell death. Thus, cancer cell may have less capacity to compensate for the HDAC inhibitor effects than normal cells [28].

In (Fig. 6D), Chk2 phosphorylation is known to be occurred by ATM dependent manner in response to IR [Ref.^2^], however, phospho-Chk2 was more increased in cells combination treated with IR and HDAC2 siRNA than those in IR alone treatment, ATM-independently. Therefore, selective depletion of HDAC2 would be sufficient to potentiate Chk2 phosphorylation and confer sensitivity to DNA damage. Although further study is needed to identify the factor responsible for phosphorylation of Chk2 induced by inhibition of HDAC2, our study may provide insight into the mechanism by which HDAC inhibitors potentiate radiotherapy and may provide guidance in the further development of therapeutic agents that more selectively inhibit HDAC2.

In conclusion, (Fig. 6F) depicts our proposed scheme in which SAHA or HDAC2 siRNA treatment of lung cancer cells results in Mdm2 downregulation and p53 activation, consequently downregulation of survivin. Downregulation of survivin enhances the responsiveness of the cells to ionizing radiation, then rendering the tumor cells less resistant to ionizing radiation-induced cell death.

## MATERIAL AND METHODS

### Cell cultures and reagents

A549, H1299 and H460 human lung cancer cells purchased from the American Type Culture Collection (Manassas, VA, USA), Lu99 human lung cancer cells, purchased from the RIKEN cell bank (Tsukuba, Japan), and HCT 116 colorectal cancer cells (p53 null and p53 wild) were supplied by Dr. Kee-Ho Lee (KIRAMS, KOREA) were grown in the recommended growth medium (Invitrogen, Carlsbad, CA, USA). SAHA was purchased from ALEXIS Corporation (Lausen, Switzerland). Antibodies against HDAC1, HDAC2, HDAC3, cIAP2, Mdm2, HA, Myc and β-actin were acquired from Santa Cruz Biotechnology (Santa Cruz, CA, USA). HDAC4, SIRT1, SIRT2, histone 3, acetyl-histone 3, acetyl-histone 4, acetyl-p53 (Lys382), puma, ubiquitin, caspase 3, cleaved PARP, p-ATM, ATM, p-ATR, ATR, p-Chk1, Chk1, p-Chk2, Chk2, p-γH2AX, γH2AX and survivin antibodies were acquired from Cell Signaling Technology (Beverly, MA, USA). XIAP, caspase 7 and p21 antibodies were purchased from BD Biosciences Pharmingen (San Diego, CA, USA), and the p53 antibody was from Novocastra Lab. Ltd. (Newcastle, UK). The Flag antibody, Nutlin-3A and MG132 were from Sigma. (St Louis, MO, USA). The siRNAs targeting HDAC1, HDAC2, HDAC3, or HDAC4 were from Santa Cruz Biotechnology. Two different HDAC2 siRNAs (siHDAC #2 and siHDAC #3) and p53-specific siRNA were purchased from Ambion (Austin, TX, USA).

### Transfections and treatments

A549 cells in 1 ml of serum-free medium were transfected with plasmid (0.1-0.2μg) or siRNA (50-120 nM) for 4 h at 37°C in a CO_2_ incubator using Lipofectamine (Invitrogen) as described by the manufacturer. The media were then replaced with fresh media containing 10% fetal bovine serum and cells were incubated for an additional 2 h. After transfection, cells were treated with SAHA and/or IR and analyzed as described below. The cells were irradiated using a ^137^Cs γ-ray source (Atomic Energy of Canada Ltd., Canada).

### Reverse transcription-polymerase chain reaction (RT-PCR)

Total RNA was isolated using a RNA mini kit (Qiagen, Valencia, CA, USA). An aliquot of total RNA (2 μg) was transcribed into cDNA using an RT^2^ First Strand kit (Qiagen). cDNA was amplified with *Taq* polymerase (Promega, Madison, WI, USA) using the specific primer pairs (Santacruz) for conventional PCR. For qPCR, cDNA was amplified with a KAPA SYBR FASR qPCR kit (Kapa Biosystems, Woburn, MA, USA) using the specific primer pairs (Origene Technologies, Rockville, MD, USA).

HDAC2 and survivin mRNA expression levels in lung cancer patient tissue were analyzed using a TissueScan Cancer Array from Origene Technologies, according to the manufacturer's protocols. In brief, after aliquot 25 μL of the PCR pre-mix including β-actin or HDAC2 specific primer pairs to each well (Tissue cDNAs of each array are synthesized from high quality total RNAs of pathologist-verified tissues), the thermocycling was performed. The condition was followed: pre-soak 95°C for 10 min and 39 cycles of 95°C for 15 s, 60°C for 20 s.

### Western blotting

Cells were harvested and lysed in RIPA buffer (50 mM Tris-HCl pH 7.5, 150 mM NaCl, 1% Nonidet P40, 0.5% sodium deoxycholate, and 0.1% SDS) supplemented with a protease/phosphatase inhibitor cocktail (Roche, Mannheim, Germany). Equal amounts of protein (20-50 μg) were separated by SDS-PAGE and transferred to a nitrocellulose membrane. Membranes were blocked by incubating with 3% skim milk in Tris-buffered saline (TBS) for 1 h and then were incubated overnight with the appropriate primary antibodies (1000:1). Membranes were then incubated with HRP-conjugated secondary antibody (3000:1) for 1 h. Immunoreactive proteins were visualized using enhanced chemiluminescence reagents (Amersham Biosciences, Little Chalfont, UK).

### Measurement of cell viability

Cell viability was determined by measuring the mitochondrial conversion of 3-(4,5-dimethylthiazolyl-2)-2,5-diphenyltetrazolium bromide (MTT) to a colored product. Cells were treated as indicated, and the medium was exchanged with serum-free medium containing 1 mM MTT. After 2 h of incubation at 37°C, cells were solubilized in DMSO. The amount of formazan, the converted form of MTT, was determined by measuring absorbance at 595 nm.

### Clonogenic assay

Cells were transfected with 60 nM siRNA and incubated for 24 h. Transfected cells were seeded at a concentration of 300-500 cells/60-mm dish. After 24 h, cells were irradiated with different doses (1 or 2 Gy) of IR (γ-irradiation). After culturing for 18 d, colonies were stained using a Diff Quik kit (Sysmex, Kobe, Japan), and the number of colonies greater than 2 mm in diameter were counted. The surviving fraction was calculated by dividing the number of colonies in treated cell groups by that in the control group.

### Ubiquitination assay

Cells were lysed in lysis buffer (20 mM Tris pH 7.5, 150 mM NaCl, 1 mM EDTA, 1 mM EGTA, 1% Triton X-100 and protease/phosphatase inhibitor cocktail) and centrifuged at 12000 rpm for 20 min. Cell lysates were immunoprecipitated using an anti-Mdm2 or anti-Flag antibodies and protein A/G agarose beads overnight at 4°C. The beads were washed with Tris-Cl buffer and boiled in 2x SDS sample buffer. Proteins in the supernatant were separated by SDS-PAGE and analyzed by immunoblotting using an anti-ubiquitin or anti-HA antibodies as described [23].

### Statistical Analysis

*P* values were calculated by applying the two-tailed Student's test to data from independent experiments. Quantitative data are presented as means ± standard deviation (SD).

## SUPPLEMENTARY MATERIAL FIGURES




