# Supplementary figures and images for: Selective inhibition of histone deacetylase 2 induces p53-dependent survivin downregulation through MDM2 proteasomal degradation
Source: Oncotarget. 2014 Dec 31;6(28):26528–40. doi: 10.18632/oncotarget.3100 (PMC4694920; doi:10.18632/oncotarget.3100)

**Fig.1A**

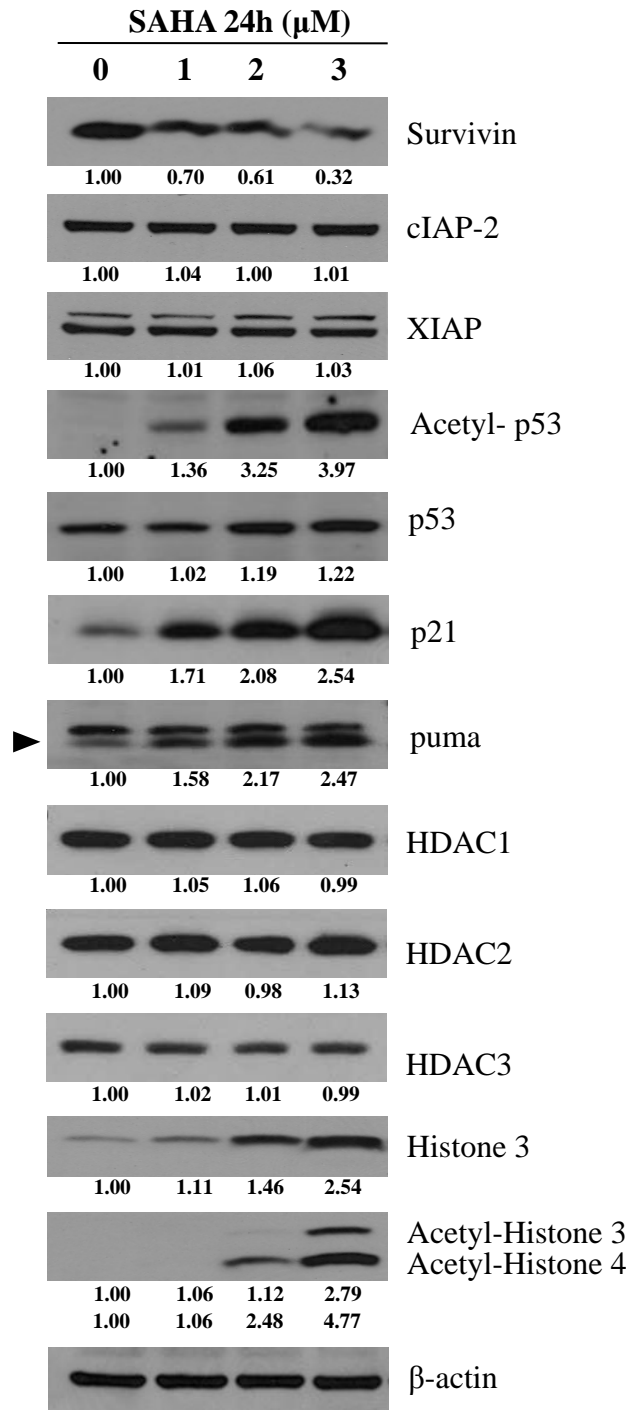

**Fig.1C**

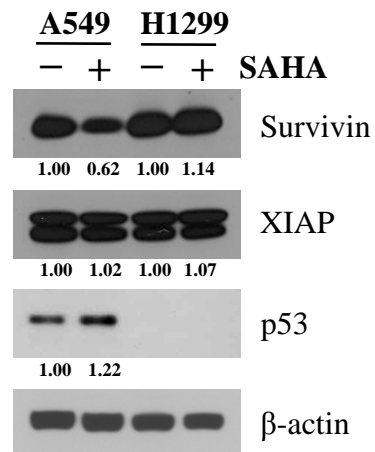

**Fig.1D**

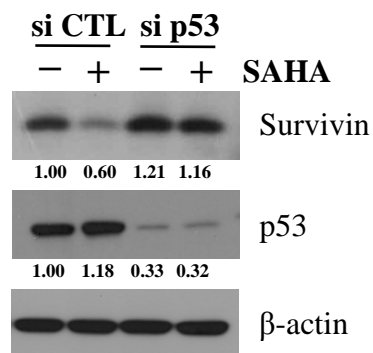

**Fig.1E**

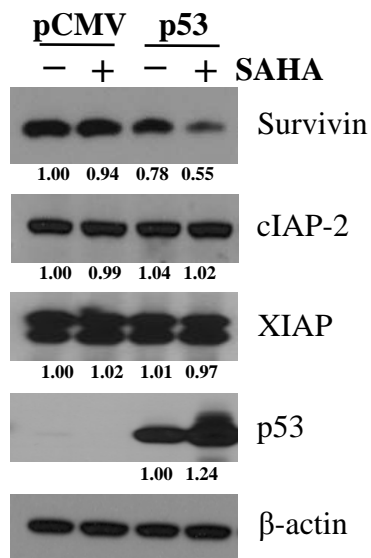

**Fig.1G**

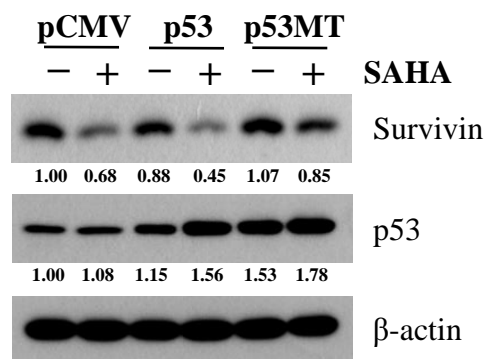

**Fig.1F**

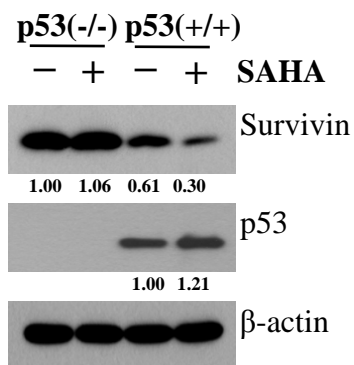

**Fig.2A**

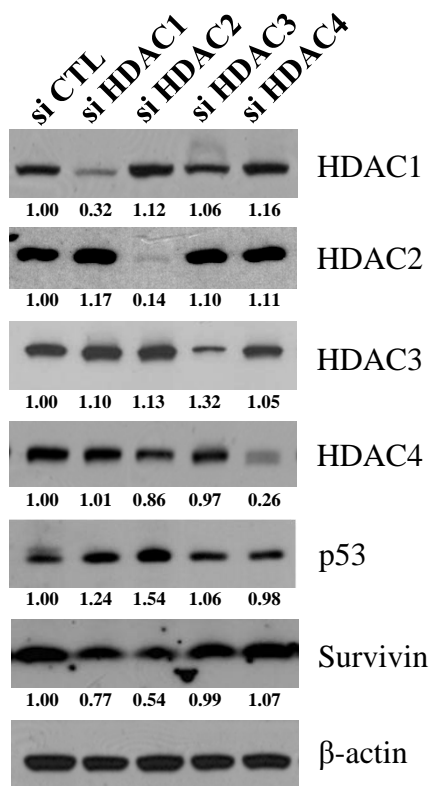

**Fig.2C**

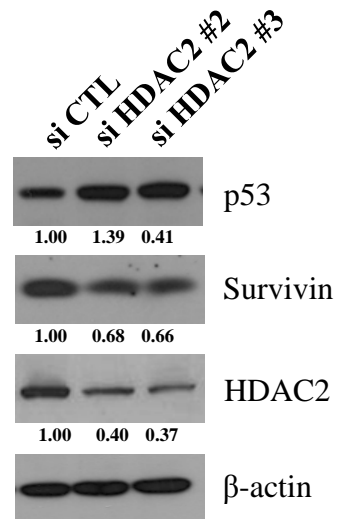

**Fig.2B**

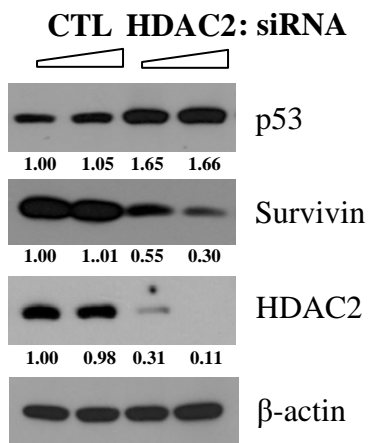

**Fig.2D**

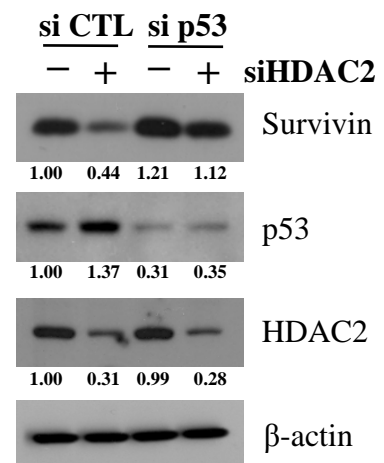

Supplement: Supplementary file 1 [file oncotarget-06-26528-s001.pdf]

**Fig.3A**

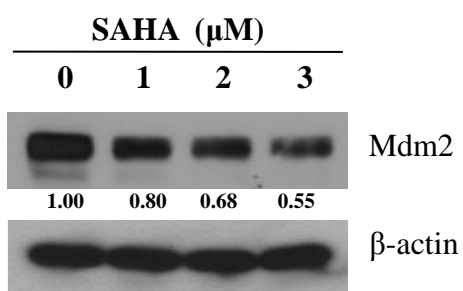

**Fig.3D**

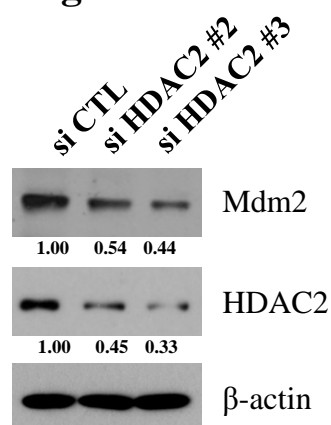

**Fig.3B**

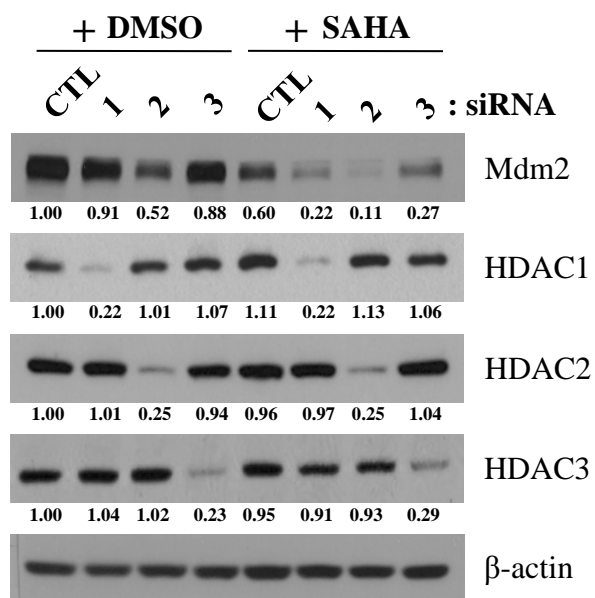

**Fig.3C**

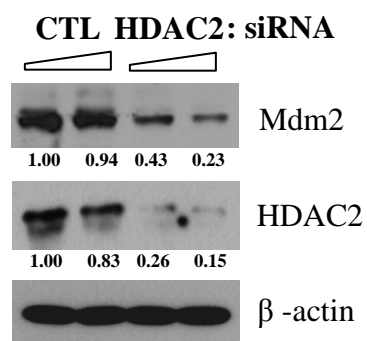

**Fig.5A**

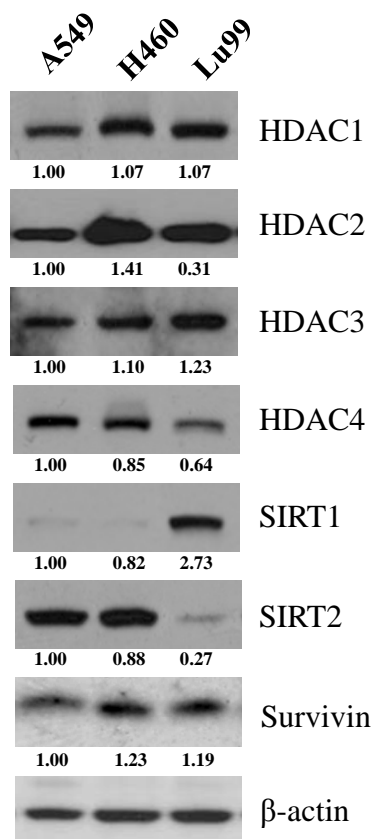

**Fig.6C**

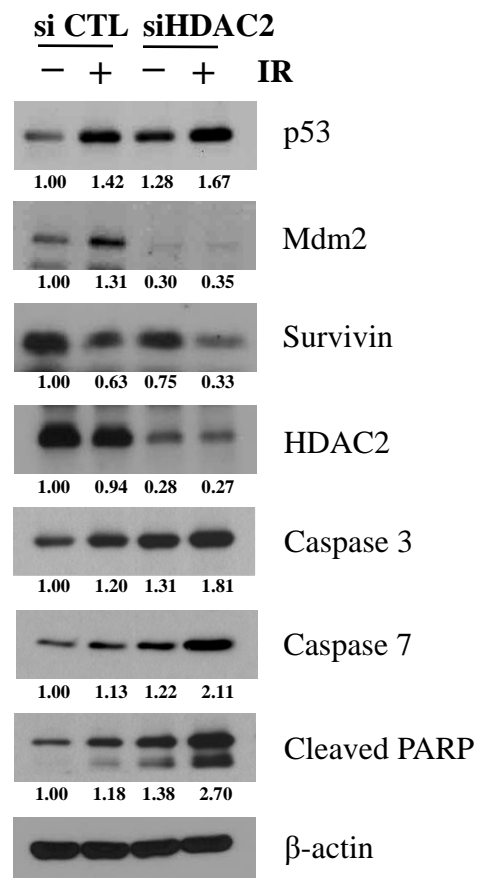

**Fig.6D**

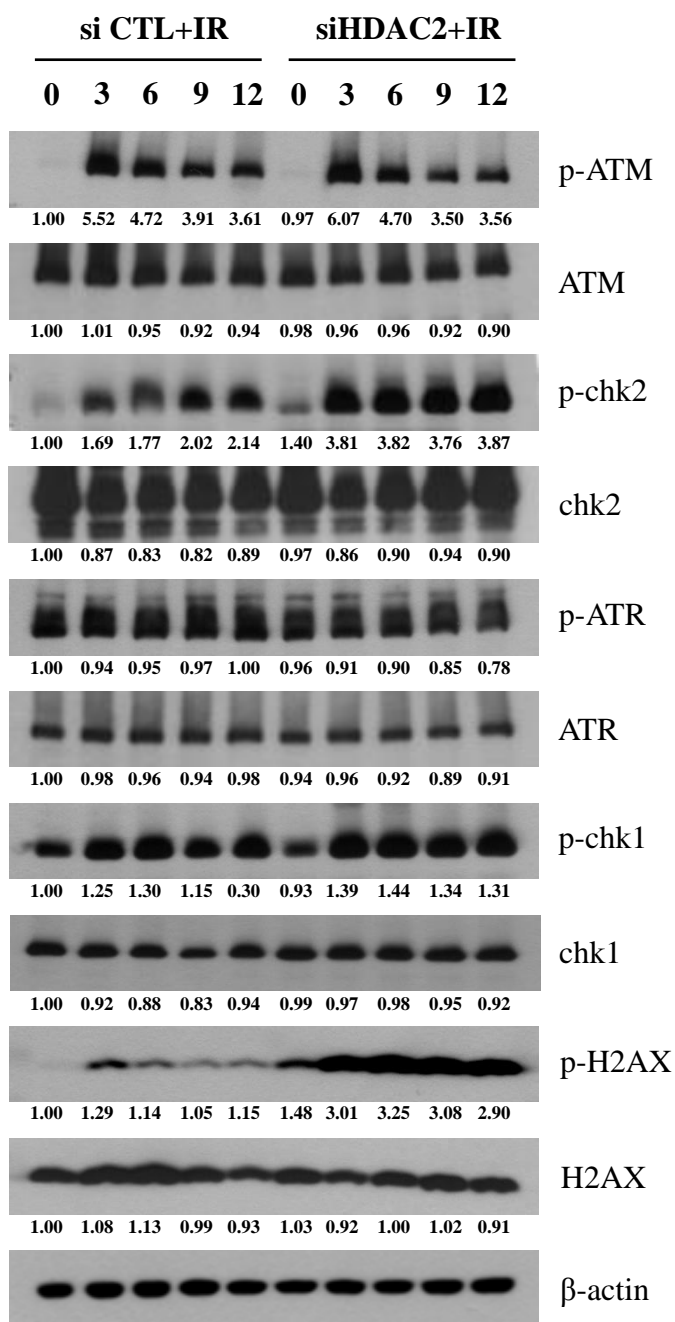

Supplement: Supplementary file 2 [file oncotarget-06-26528-s002.pdf]
